# Supplementary material for: A balance between aerodynamic and olfactory performance during flight in Drosophila
Source: Nat Commun. 2018 Aug 10;9:3215. doi: 10.1038/s41467-018-05708-1 (PMC6086917; doi:10.1038/s41467-018-05708-1)
Supplement: Supplementary file 1 — Supplementary Information [file 41467_2018_5708_MOESM1_ESM.pdf]

Supplementary Information for

A balance between aerodynamic and olfactory performance during flight in *Drosophila*

Li et al.

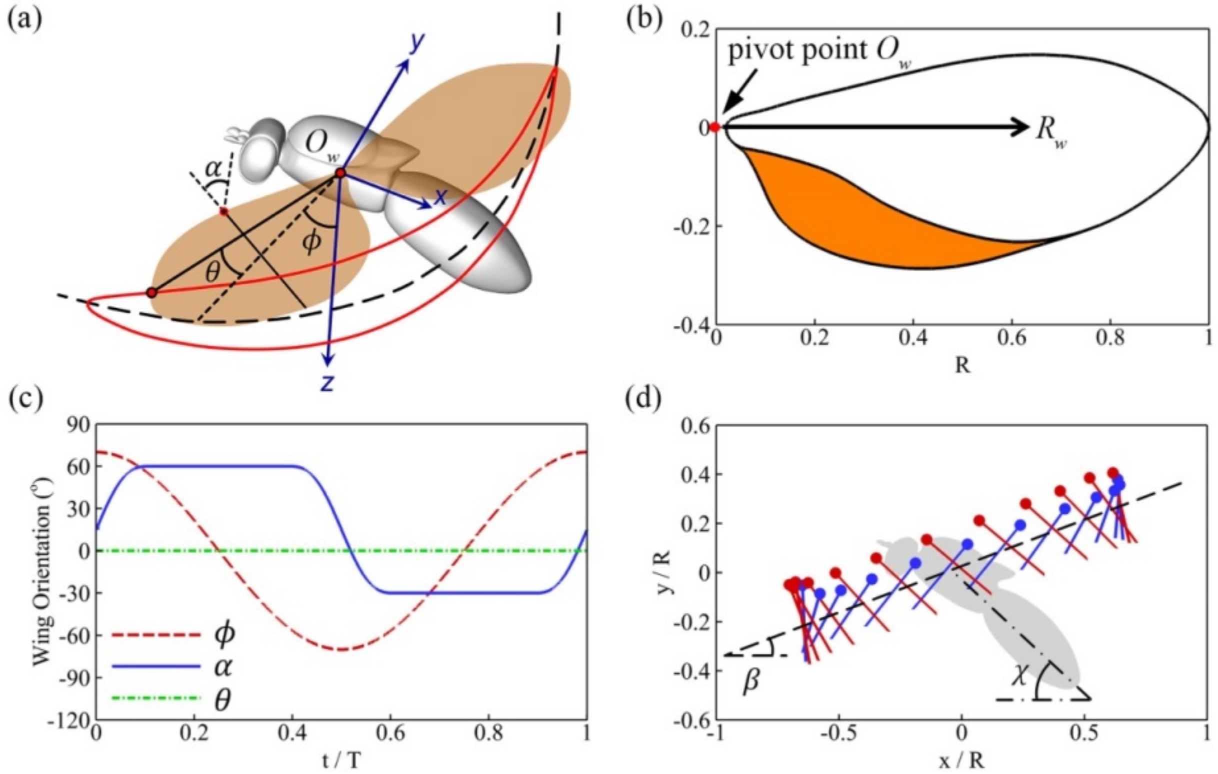

Supplementary Figure 1 Fruit fly model. (a) The fruit fly *D. melanogaster* model, with the definition of wing Euler angles. (b) The original and modified wings: the geometric difference is shown in orange. The wings rotate around the pivot points (O_w) within the stroke plane. (c) Time course of wing Euler angles. (d) Wing chord kinematics in an entire flapping cycle within the stroke plane ($\beta = 20°$). The small circle at each wing segment indicates the leading edge. The red and blue lines represent wing chord at 75% wingspan during downstroke and upstroke, respectively. The entire body is inclined by $\chi = 45°$ with respect to the horizontal plane.

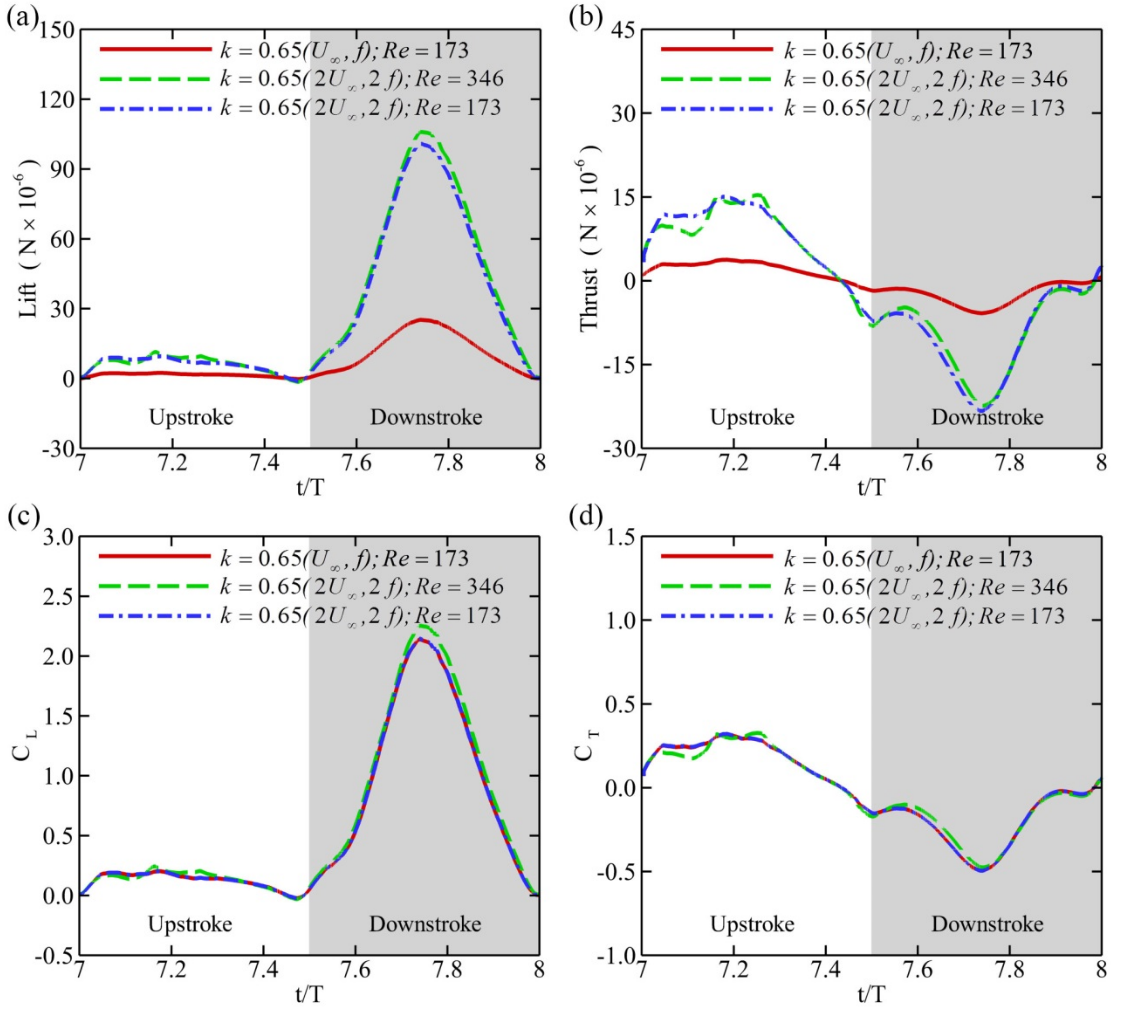

Supplementary Figure 2 Forces and non-dimensional force coefficients with matched reduced frequency (k). (a) Lift force. (b) Thrust force. (c) Non-dimensional Lift coefficient (C_L). (d) Thrust coefficient (C_T). The red solid curves are the case presented in the main text, with U_∞ = 0.94 m/s, f = 213 Hz, and k = 0.65. By doubling forward flight speed (U_∞) and flapping frequency (f), the reduced frequency of the green dashed curve is the same as the red solid curve but with a Reynolds number twice as high. If we also keep the Reynolds number as the same as the original case (red solid) by adjusting the kinematic viscosity in the simulation, the blue dashed-dot curve is identical to the red solid cure for nondimensionalized C_L and C_T, although the effect of *Re* is much smaller than the effect of *k*. This demonstrates that the results of the current study may be extrapolated to other forward speeds based on normalized analysis of matching reduced frequencies and Reynolds number.

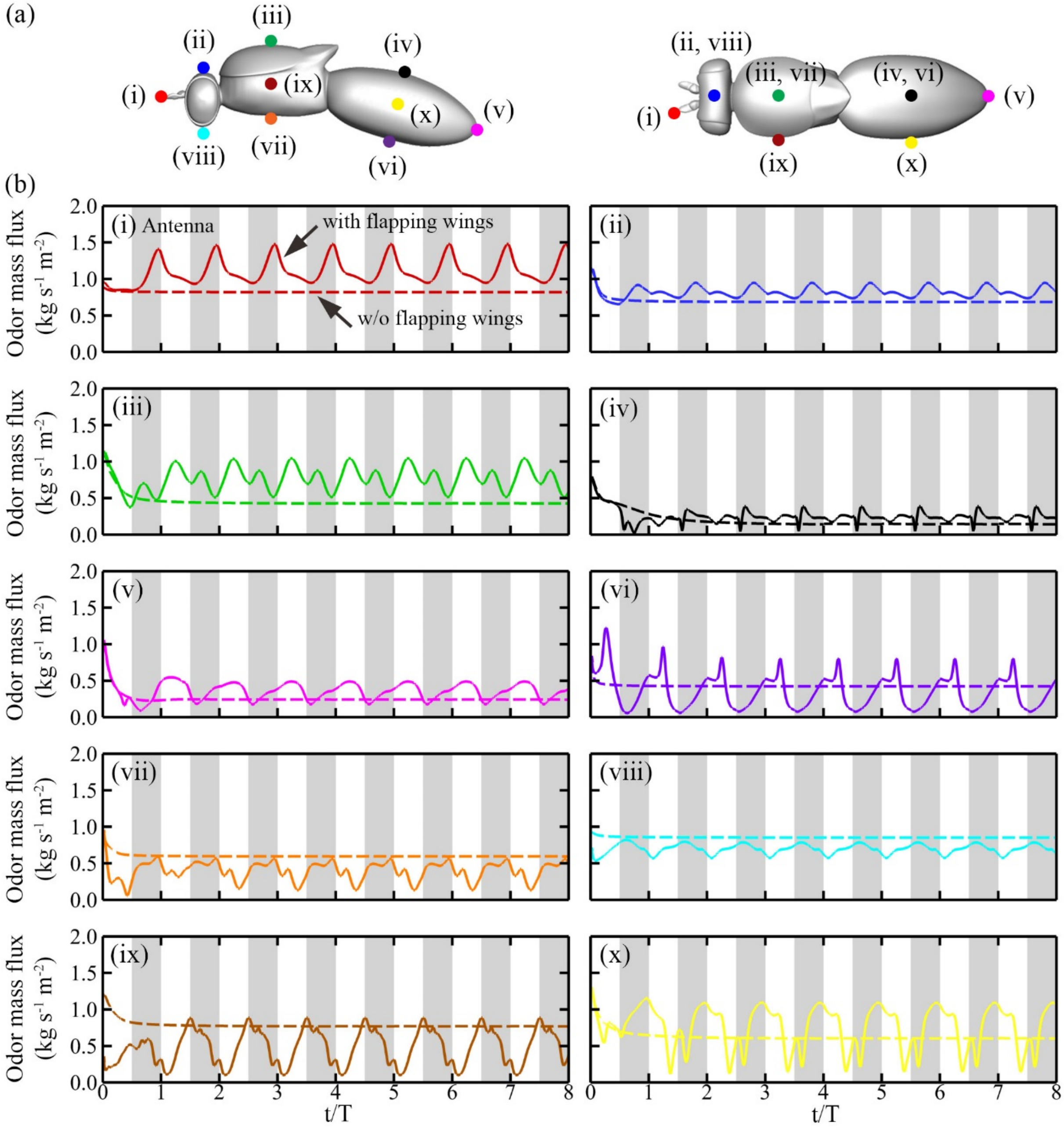

Supplementary Figure 3 Odor mass flux at 10 different locations around fruit fly body during forward flight at *k*=0.65 and *Re*=173. (a) The probe location for measuring the odor mass flux around fruit fly body. (b) Time history of odor mass flux at 10 different locations. The shaded areas represent downstrokes. The dashed lines indicate the odor mass flux without wings flapping.

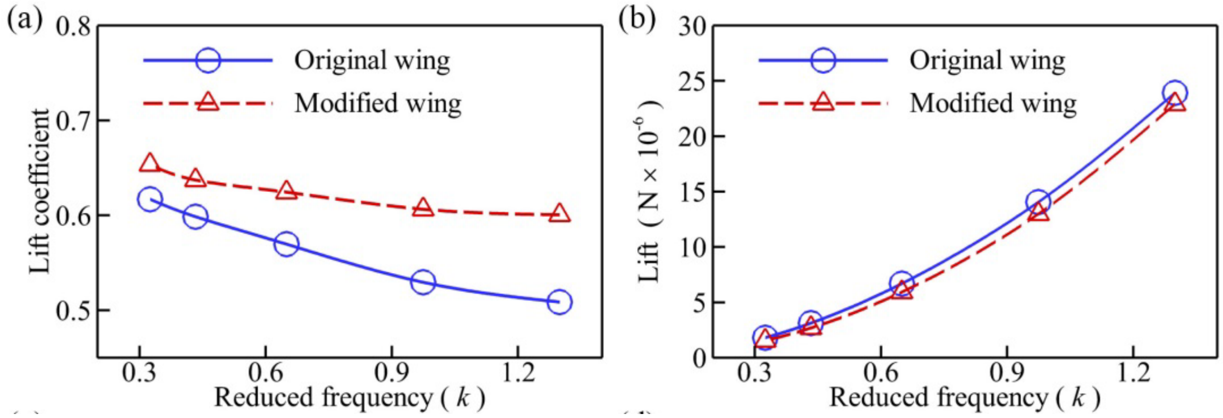

Supplementary Figure 4 Comparison of the force generation between original wing and modified wing that has 20% reduction in surface area. Cycle-averaged lift coefficient (a) and lift force (b) as function of the reduced frequency.

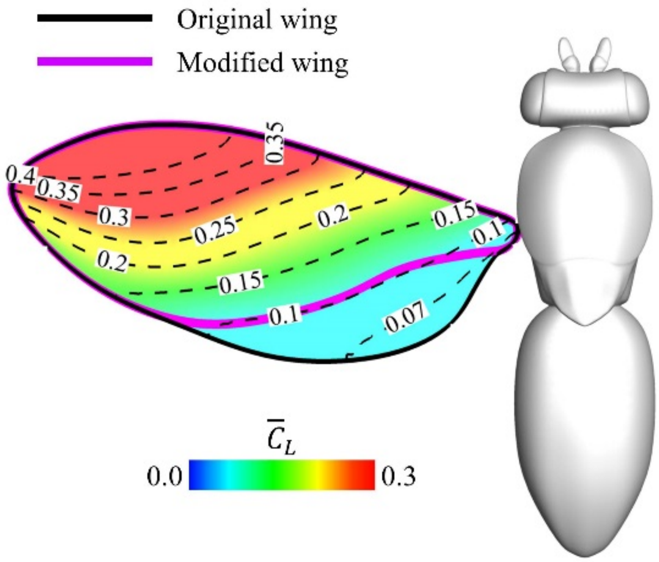

Supplementary Figure 5 The clipping line of the modified wing is based on the surface contour of the cycle-average lift coefficient (baseline case, k = 0.65) at the level of 0.1.

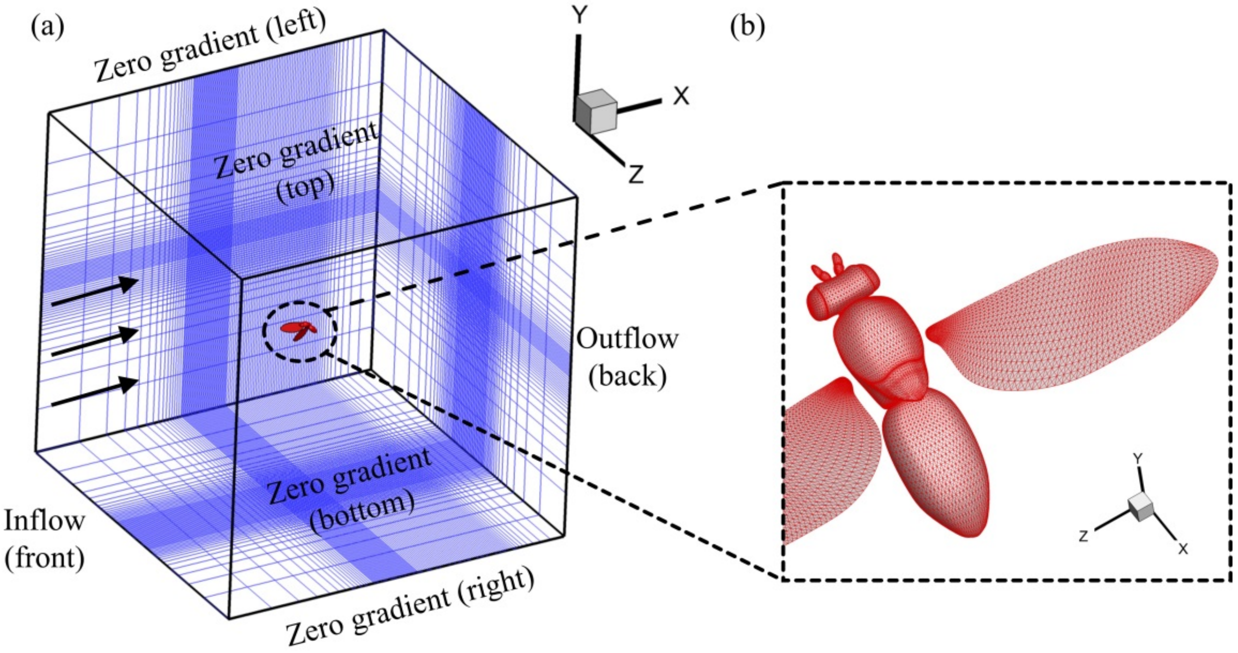

Supplementary Figure 6 Simulation Setup of a fruit fly in forward flight. (a) Schematic of the computational mesh (approximate 10 million grids) and boundary conditions employed in the current simulations. (b) High-density surface mesh with approximately 29,000 and 5,000 triangular elements was used to define the body and each wing, respectively.

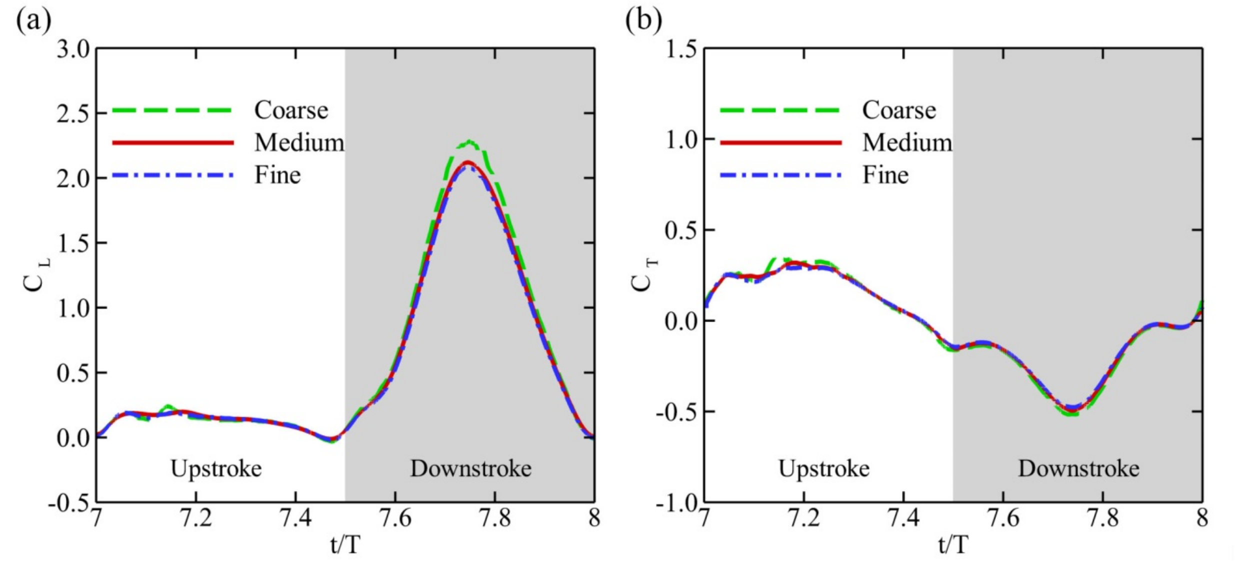

Supplementary Figure 7 Comparison of lift (a) and thrust (b) coefficients to demonstrate grid independence of the computed results. The grids employed in the simulations were $193\times129\times193 = 4.8\times10^6$ (for coarse mesh), $289\times137\times249 = 9.9\times10^6$ (for medium mesh), and $353\times161\times321 = 18.2\times10^6$ (for fine mesh). The difference of lift and thrust peaks between the medium grid (presented in this article) and fine grid is less than 2.1% and 0.9%, respectively. This demonstrates that the results are grid independent. Thrust coefficient has positive and negative values, indicating that the fruit fly produced thrust in the upstroke and drag in the downstroke. The cycle-averaged Thrust coefficient is close to zero (~ 0.018). Thus, the force balance is approximately achieved in the horizontal direction, an indication of self-propelled forward flight.

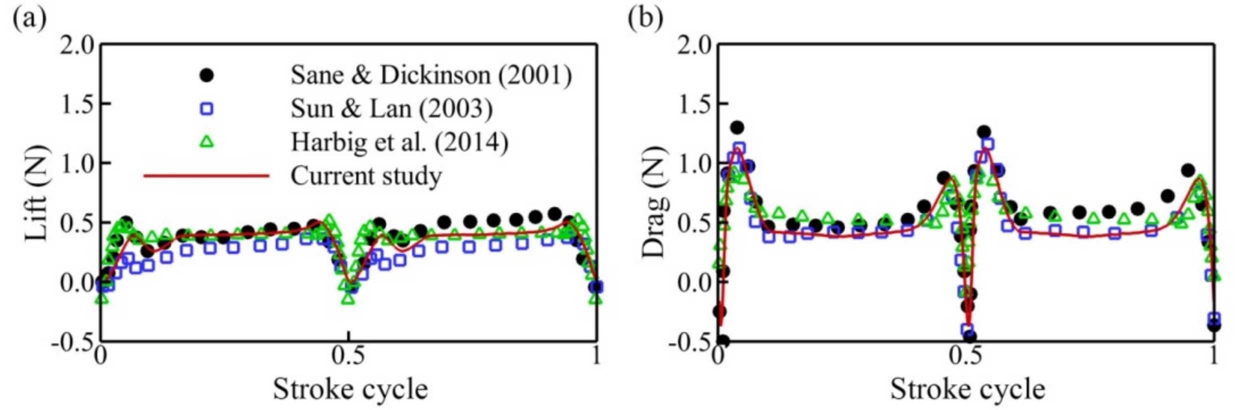

Supplementary Figure 8 Benchmark of the 3D flapping wing simulation. Comparison of lift (a) and drag (b) from the current CFD solver with the experimental results of Sane and Dickinson [1] and the computational results of Sun and Lan [2] and Harbig et al. [3] over a flapping cycle. The stroke amplitude is 180°, and the angle of attack at the midstroke is 50°.

Supplementary Table 1 Wing flapping frequencies and mean wing-tip velocities at various reduced frequencies.

k	f (Hz)	$\bar{U}_{tip}$ ($m\ s^{-1}$)
0.33	107	1.51
0.43	160	2.01
0.65	213	3.02
0.97	320	4.53
1.30	426	6.04

Supplementary References

1. Sane SP, Dickinson MH. The control of flight force by a flapping wing: lift and drag production. *J. Exp. Biol.* **204**, 3401-3401 (2001).

2. Sun M, Wu JH. Aerodynamic force generation and power requirements in forward flight in a fruit fly with modeled wing motion. *J. Exp. Biol.* **206**, 3065-3083 (2003).

3. Harbig R, Sheridan J, Thompson M. The role of advance ratio and aspect ratio in determining leading-edge vortex stability for flapping flight. *J. Fluid Mech.* **751**, 71-105 (2014).
