# Supplementary material for: A balance between aerodynamic and olfactory performance during flight in Drosophila
Source: Nat Commun. 2018 Aug 10;9:3215. doi: 10.1038/s41467-018-05708-1 (PMC6086917; doi:10.1038/s41467-018-05708-1)
Supplement: Supplementary file 3 — Description of Additional Supplementary Information [file 41467_2018_5708_MOESM3_ESM.pdf]

Description of Additional Supplementary Files

File Name: Supplementary Movie 1
Description: Fruit fly in forward flight. The movie shows wing and body modeling of a fruit in forward flight, kinematics and simulation setup, and visualization of odor plume structure (Lagrangian tracking) throughout the flapping cycles.
